# An integrated approach for the benthic habitat mapping based on innovative surveying technologies and ecosystem functioning measurements

**DOI:** 10.1038/s41598-024-56662-6

**Published:** 2024-03-11

**Authors:** Daniele Piazzolla, Sergio Scanu, Francesco Paolo Mancuso, Mar Bosch-Belmar, Simone Bonamano, Alice Madonia, Elena Scagnoli, Mario Francesco Tantillo, Martina Russi, Alessandra Savini, Giorgio Fersini, Gianluca Sarà, Giovanni Coppini, Marco Marcelli, Viviana Piermattei

**Affiliations:** 1 CMCC Foundation - Euro-Mediterranean Center on Climate Change, Lecce, Italy; 2 Laboratory of Ecology, Department of Earth and Marine Sciences (DiSTeM), University of Palermo, 90123 Palermo, Italy; 3 NBFC, National Biodiversity Future Center, Spoke 1, 90133 Palermo, Italy; 4 Laboratory of Experimental Oceanology and Marine Ecology, Department of Ecological and Biological Sciences DEB, University of Tuscia, Port of Civitavecchia, 00053 Civitavecchia, Italy; 5 Department of Earth and Environmental Sciences (DISAT), University of Milano-Bicocca, 20126 Milan, Italy; 6 Port Authority System of the Central Northern Tyrrhenian Sea, 00053 Civitavecchia, Italy

**Keywords:** Ecology, Environmental sciences, Ocean sciences

## Abstract

Among marine ecosystems globally, those in the Mediterranean Sea, are facing many threats. New technologies are crucial for enhancing our understanding of marine habitats and ecosystems, which can be complex and resource-intensive to analyse using traditional techniques. We tested, for the first time, an integrated multi-platform approach for mapping the coastal benthic habitat in the Civitavecchia (northern Latium, Italy) coastal area. This approach includes the use of an Unmanned Surface Vehicle (USV), a Remote Operated Vehicle (ROV), and in situ measurements of ecosystem functionality. The echosounder data allowed us to reconstruct the distribution of bottom types, as well as the canopy height and coverage of the seagrass *Posidonia oceanica*. Our study further involved assessing the respiration (Rd) and net primary production (NCP) rates of *P. oceanica* and its associated community through in situ benthic chamber incubation. By combining these findings with the results of USV surveys, we were able to develop a preliminary spatial distribution model for *P. oceanica* primary production (PP-SDM). The *P. oceanica* PP-SDM was applied between the depths of 8 and 10 m in the studied area and the obtained results showed similarities with other sites in the Mediterranean Sea. Though in the early stages, our results highlight the significance of multi-platform observation data for a thorough exploration of marine ecosystems, emphasizing their utility in forecasting biogeochemical processes in the marine environment.

## Introduction

The coastal marine environment is frequently impacted by various human activities, stemming from different resource uses. These uses often clash with each other, leading to significant impacts on the ecosystem functioning of coastal marine habitats^1^.

*Posidonia oceanica* (L.) Delile meadows are one of the most important and productive habitats in the coastal areas of the Mediterranean Sea and are considered as a key coastal structuring habitat enhancing biodiversity levels^2^. Because of their ecological importance, *P. oceanica* meadows have been included in Annex 1 of the European Union (EU) Directive 92/43/EEC as a priority habitat^3^. *Posidonia oceanica* plays a key role in carbon dioxide fixation and organic carbon production in coastal euphotic zones^4^, provides habitat, food, and nurseries for marine organisms [e.g.,^5–7^], stabilizes the seafloor, and protects the coast from erosion processes [e.g.,^8^].

Primary production (PP) in *P. oceanica* displays notable variability across depths and temporal scales, encompassing sub-daily, seasonal, and year-to-year fluctuations^9,10^. This variability can arise from natural factors such as variations in incident solar radiation due to cloud cover or seasonality, as well as human-induced stressors. Authors in^11^ propose that high-frequency monitoring of PP could provide insights into the health status and long-term evolution of *P. oceanica* meadows. Moreover, inter-annual variations in PP in response to extreme weather events offer natural experiments for understanding ecosystem responses to future global and climate changes. Most techniques used to measure PP primarily focus on processes occurring within the water column but lack differentiation of the processes involved between the air–water and water–sediment interfaces. To obtain more information on O_2_ or CO_2_ gas exchange between water and sediment layers, benthic chambers can be employed. These chambers involve enclosing an area of meadow and associated sediment within clear (and dark) chambers. Artifacts associated with light and flow can be minimized by using specific chambers designed to reduce light attenuation, employing stirrers to create flow, and keeping incubation times short (< 30 min)^12^.

The proximity of *Posidonia oceanica* meadows to coastal regions renders them highly susceptible to human activities. This has led to a widespread decline in these seagrass beds across the Mediterranean Sea, as evidenced by numerous studies. This situation underscores the urgent need for scientists to deepen their understanding of how anthropogenic pressures influence ecosystem functionality. Central to this effort is the enhancement of our capabilities to map and monitor submerged marine habitats. Although this task is essential, it presents significant challenges, notably more complex than those encountered in terrestrial ecosystems, primarily due to the substantial costs associated with data collection. To effectively assess the condition of submerged habitats and anticipate the impact of human disturbances, it is imperative to develop sustainable observation systems. These systems should incorporate cost-effective technologies, enabling extensive data acquisition and facilitating the integration of diverse data types^13^. However, the accessibility of user-friendly, low-cost marine monitoring instrumentation currently remains a limiting factor^14^. Additionally, effective observing systems should incorporate specific measurement platforms capable of providing adequate spatiotemporal resolution and coverage to meet the diverse needs of monitoring^15^. Only a small portion of the seafloor is mapped at a resolution similar to terrestrial landscapes, making marine ecosystems less described than their terrestrial counterparts. This results in significant information gaps at various spatial and temporal scales, especially at the local level where data from different studies using diverse methodologies are challenging to compare.

Worldwide, biotope/habitat mapping and coastal monitoring activities involve the help of scientific operators to take environmental samples for laboratory analysis as well as the use of technologies such as echosounders (single beam, multi beam, and side scan sonar) [e.g.,^16–21^] and Remote Operated Vehicles [ROVs; e.g.,^22–24^]. However, sampling activities and traditional marine monitoring with fuelled vessels increase survey costs and pollution risks. In this context, the development of new monitoring methodologies involving the use of innovative autonomous platforms is of particular help, contributing to the reduction of work costs, while enabling rapid data collection and facilitating measurements in hard-to-reach sites (e.g., Marine Protected Areas—MPAs, extremely shallow waters, areas forbidden to ordinary navigation) [e.g.,^25–28^]. Effective new approaches involve the use of Unmanned Surface Vehicles (USVs) equipped with specific sensors for mapping purposes^29^, Uncrewed Aerial Systems (UAS) equipped with light weight sensors (e.g., multispectral, hyperspectral, and LiDAR systems) [e.g.,^30,31^], and technologies to support divers' scientific activities (for example, Diver Propulsion Vehicles—DPVs) [e.g.,^32^]. Furthermore, the joint use of these technologies improves operational efficiency, data acquisition and resolution^33,34^.

Here we report the results of the coastal benthic habitats mapping along the coast of Civitavecchia (Northern Latium, Italy) using an integrated approach mainly based on USV, ROV technologies, and ecosystem functioning measurements (PP of *P. oceanica* meadows). The aim is to demonstrate the effectiveness of an integrated approach for local-scale investigations, providing detailed information for ecosystem management and conservation actions. For the first time, we present a spatial distribution model (PP-SDM) for *P. oceanica* meadows in the northern Latium coastal area. Understanding habitats at a small scale in this area is crucial to disentangle the potential effects of the large number of industrial activities, such as the Port of Civitavecchia, which is undergoing expansion works with potential impacts on nearby coastal ecosystems.

## Results

**Figure 1:**
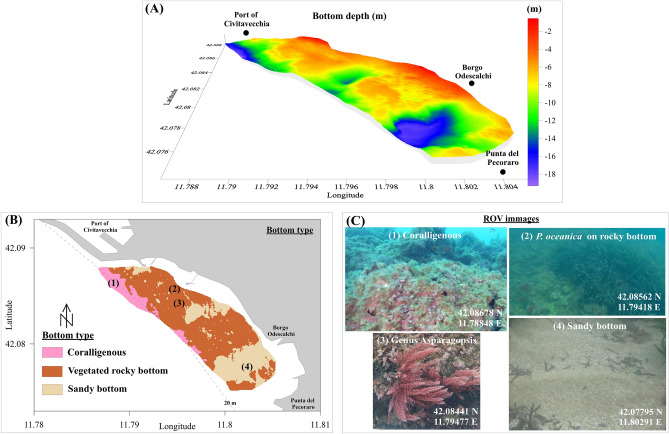
Bottom depth (**A**), bottom type (**B**), and ROV images of substrates (**C**). Each of the ROV images shows the geographic coordinates of the acquisition site.

The seabed showed heterogeneous morphology and substrate types. The sea area closest to the Port of Civitavecchia showed a gradual increase in depth starting from the shoreline up to 10 m (this area extended about 400 m offshore), followed by a more abrupt depth increase up to 20 m within a 200 m span. The southernmost area generally had a shallow seabed (below 10 m), with two deeper areas observed at the northern boundary of the survey plan (12–15 m deep, 300 m from the shoreline) and near Punta del Pecoraro (up to 20 m deep) (Fig. 1A).

The seabed composition predominantly featured vegetated rocky bottoms, with sandy bottoms and Coralligenous bioconstructions present to a lesser extent (Fig. 1B and C). Coralligenous bioconstructions were found over a depth of 12 m, while most of the sandy bottom was located south of Borgo Odescalchi, corresponding to the presence of an inland ditch. The presence of sands resulted from the deposition process of suspended sediments in this depression area due to local coastal dynamics. This seabed morphology is characteristic of the coastal area between the mouth of the Mignone River and Capo Linaro (Fig. 1)^35^, causing discontinuity in the distribution of benthic habitats.

Submerged vegetation was generally found throughout the investigated area, but to a lesser extent in areas with sandy bottoms located along the northern boundary of the survey plan and immediately south of Borgo Odescalchi (Fig. 1B and C).

**Figure 2:**
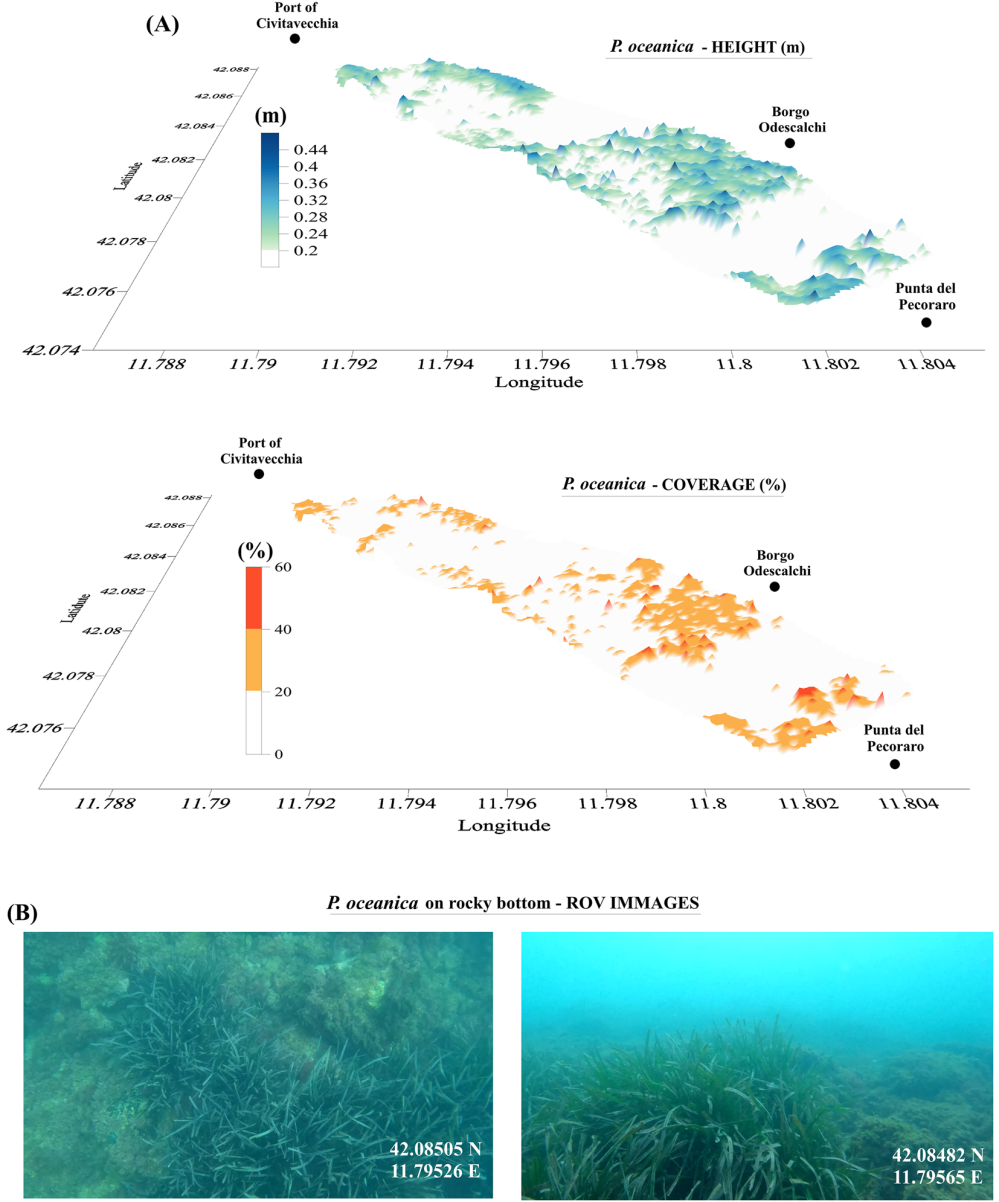
*P. oceanica* coverage (%) and canopy height (m) (**A**), and ROV images of *P. oceanica* meadows in the area (**B**). Each of the ROV images shows the geographic coordinates of the acquisition site.

*P. oceanica* presence was observed in most of the area, with a maximum coverage of 60%, a minimum canopy height of 0.21 m, a maximum canopy height of 0.48 m, and an average canopy height value of 0.3 ± 0.08 m (Fig. 2A and B). To verify the accuracy of the echosounder height measurements of *P. oceanica*, the data were compared with the canopy height measured in situ by scientific divers (blue dots, Fig. 4) and with the leaf’s length measured inside the benthic chambers (green dots, Fig. 4). The canopy height measured by divers ranged from a minimum of 0.10 m to a maximum value of 0.5 m with an average value of 0.35 ± 0.16 m. The leaf’s length measured inside the benthic chambers ranged from 0.26 m to 0.5 m with an average value of 0.38 ± 0.11 m.

A significant linear correlation was found between the height data of *P. oceanica* acquired with the echosounder and those measured by the divers (dive sites and benthic cambers) (r = 0.8, p-value < 0.05), and both datasets fitted with a linear regression model (r = 0.87, p-value < 0.05). The RMSE of the *P. oceanica* height data was 0.9 m, which corresponds to a mean error of 23%. Finally, Cohen's kappa coefficient between 0.5 and 0.6 was measured, highlighting a moderate agreement between the measurements^36^.

The in situ measurements carried out using the benthic chambers have provided important data regarding the *P. oceanica* ecosystem functioning. The Rd ranged from −48.67 mmol O_2_ m^−2^ d^−1^ to 0.82 mmol O_2_ m^−2^ d^−1^ with an average value of −21.73 ± 20.26 mmol O_2_ m^−2^ d^−1^; the NCP ranged from 1.33 mmol O_2_ m^−2^ d^−1^ to 65.33 mmol O_2_ m^−2^ d^−1^ with an average value of 34.43 ± 23.97 mmol O_2_ m^−2^ d^−1^; and the GCP ranged from 2.15 mmol O_2_ m^−2^ d^−1^ to 98.79 mmol O_2_ m^−2^ d^−1^ with an average value of 56.16 ± 42.66 mmol O_2_ m^−2^ d^−1^. Analysing the benthic chamber’s data in more detail, a significant correlation and linear regression model fit between the leaf length data and the metabolic data (Rd, NCP, and GCP) was found (leaf length vs. Rd: r = 0.83, p > 0.05; leaf length vs. NCP: r = 0.75, p > 0.05; leaf length vs. GPP: r = 0.81, p > 0.05). Finally, the equations explaining the linear regression models described above were used to obtain the spatial distribution model of Rd, NCP, and GCP of the *P. oceanica* meadow between 8 and 10 m depths.

**Figure 3:**
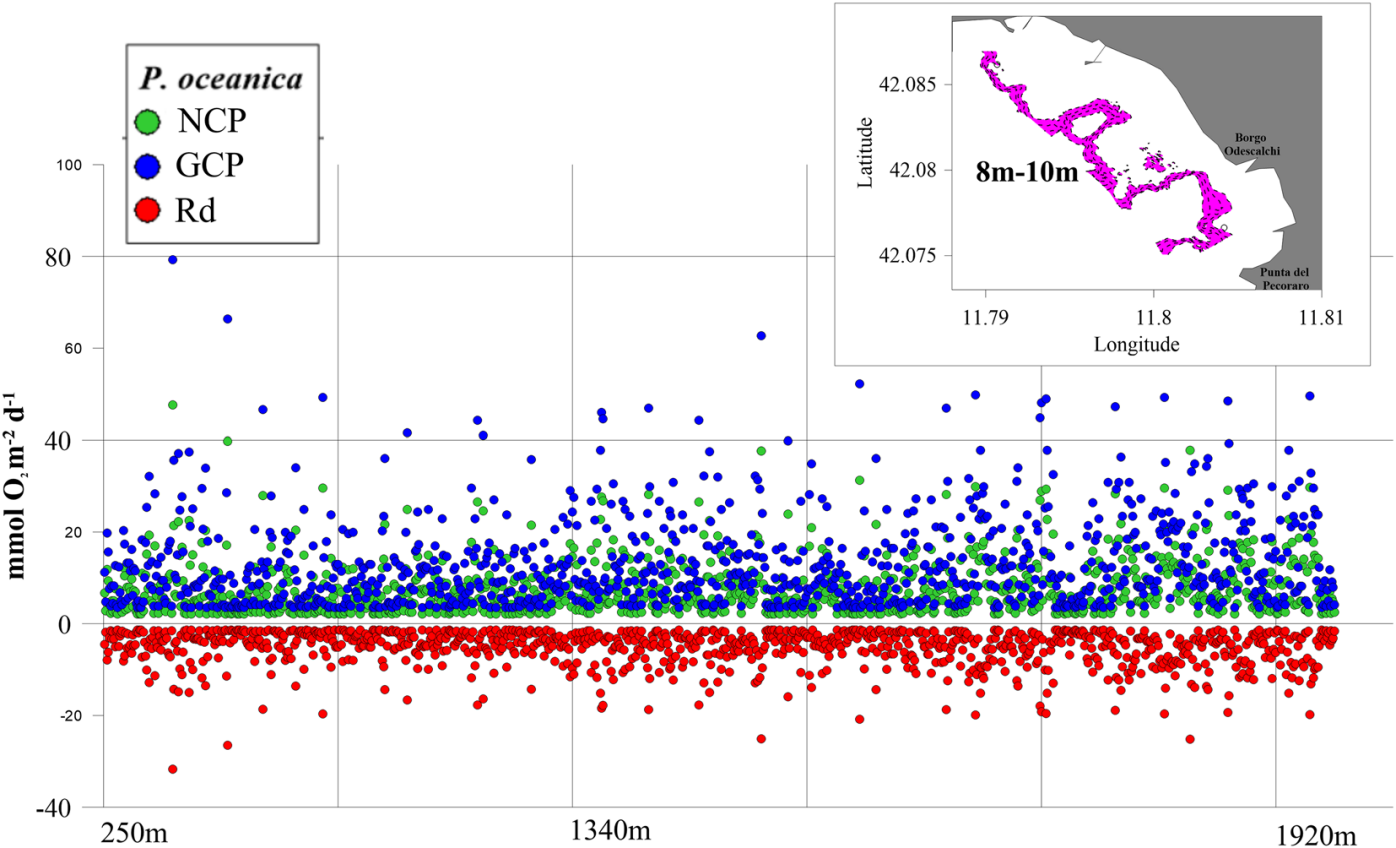
Respiration (Rd; red dots), Net Community Production (NCP; green dots) and Gross Community Production (GCP; blue dots) of *P. oceanica* between 8 and 10 m depth, from the area close to the breakwater of the Port of Civitavecchia up to a distance of 2000 m.

The preliminary *P. oceanica* PP-SDM gave very heterogeneous results (represented in Fig. 3 with respect to the distance from the Port of Civitavecchia breakwater, northwest to southeast). The modelled Rd ranged from − 31.71 mmol O_2_ m^−2^ d^−1^ to − 1.41 mmol O_2_ m^−2^ d^−1^ with an average value of − 5.12 ± 3.95 mmol O_2_ m^−2^ d^−1^; the modelled NCP ranged from 2.11 mmol O_2_ m^−2^ d^−1^ to 47.57 mmol O_2_ m^−2^ d^−1^ with an average value of 7.67 ± 5.92 mmol O_2_ m^−2^ d^−1^; and the GCP ranged from 3.52 mmol O_2_ m^−2^ d^−1^ to 79.29 mmol O_2_ m^−2^ d^−1^ with an average value of 12.79 ± 9.87 mmol O_2_ m^−2^ d^−1^.

## Discussion

The integrated approach used for mapping benthic habitats yielded reliable results, as confirmed by the values of r, RMSE, and Cohen's kappa coefficient. This approach proved particularly useful for studying substrates with non-uniform vegetation and fragmented *Posidonia oceanica* meadows. By providing rapid characterization of coastal seabed, it could be particularly useful in generating high-resolution and accurate models in studies focused on seagrass restoration activities, especially if coupled with innovative photogrammetry-based micro-bathymetry techniques^37^.

The northern Latium coast was previously studied to assess the seabed morphology using traditional survey vessels equipped with different echosounder technologies (e.g., side-scan sonar and multibeam surveys) as well as the benthic biocenoses for the ecosystem services evaluation [e.g.,^1,38–40^]. Although the use of different echosounder types allowed deep characterization of the seabed in the study area^38,40^, the seabed morphology and bottom type distribution obtained through the USV equipped with a single beam echosounder (Fig. 1) showed a substantial qualitative correspondence with these prior research works. This highlighted the utility of the proposed integrated methodology, which, compared to the use of traditional survey vessels and other echosounder technologies, was considerably less polluting, required fewer operators, was faster to execute, and more cost-effective. It could easily be applied in other coastal contexts worldwide. Furthermore, the data obtained with this integrated methodology could be used for machine learning seabed mapping and seagrasses presence and features prediction.

Carrying out winter surveys was essential to obtain a reliable distribution of the *P. oceanica* percentage cover, avoiding overestimates that could emerge from summer surveys when the long leaves could cover part of other substrates. The distribution of canopy height and percentage coverage (Fig. 2), derived from echosounder analysis, contributed to enrich the existing knowledge about seagrass meadows in the northern Latium coastal area [e.g.,^1,3,41–45^]. Moreover, these results could serve as a base layer for future investigations focused on monitoring the biogeochemical cycles of the coastal marine environment, involving several processes (e.g., PP, sea-atmosphere CO_2_ exchange) [e.g.,^46,47^] the detailed study of which is difficult to pursue using only traditional monitoring technologies or point measurements/sampling by underwater operators^29^.

The integrated use of innovative monitoring technologies and in situ surveys with benthic chambers allowed the generation of a PP-SDM (Fig. 3) of *Posidonia oceanica* meadows in the study area. The successful growth and photosynthesis of marine plants, and consequently their PP, depend on environmental conditions (e.g., light as the foremost factor, nutrients, water temperature, and salinity), and their change throughout the year^48^. Therefore, an accurate estimation of the *P. oceanica* meadows PP should take these relationships into account. The presented preliminary *P. oceanica* PP-SDM (Fig. 3) served as a snapshot of reliable values exclusively for depths between 8 and 10 m and during the winter season.

Modelled Rd, NCP, and GCP values (Fig. 3) were compared with the data reported in the literature concerning the *Posidonia oceanica* meadows PP in the Mediterranean Sea. We referred to research conducted during the last twenty years on seagrass meadows at depths ranging from 4 to 13 m, in which benthic chambers were used to incubate the plants^49–55^ (Table 1). Despite differences in the oxygen measurement methodologies, modelled Rd, NCP, and GCP values were comparable to those reported in the literature. Considering NCP, the average value for January in the Civitavecchia area (7.67 ± 5.92 mmol O_2_ m^−2^ d^−1^) was lower than the value reported by authors in^51^ for the Corsica *P. oceanica* meadows (22.93 ± 1.72 mmol O_2_ m^−2^ d^−1^) at a comparable latitude, which reflects the average of the observations obtained in the multi-annual study (around 30 years) considering exclusively the months between November and April. Lower winter values of Rd, NCP, and GCP compared to our results were reported by authors in^52^ for the island of Mallorca (Table 1). Values of GCP and Rd higher than those shown in this study were found by authors in^53^ in May 2016 in Elba Island, probably testifying to the influence of seasonality and the consequent variation of environmental factors on the PP of seagrass (Table 1).

**Table 1:** Comparison of the modelled values of winter PP indicators of *P. oceanica* in the Civitavecchia area with those reported in the literature for other areas of the Mediterranean Sea, at comparable depths; *refers to the depth of the seagrass meadow chosen for incubation through benthic chambers; **Gross Primary Production; ***Net Primary Production; **** Respiration Rate; *****original values was expressed as g m^−2^ d^−1^; ******values of two days of measurements; *******values extracted from the graphs reported in Fig. 3 in the work Ward et al. (2022).

Location	Month, year/ Season, year	Depth (m)*	Indicators	mmol m^−2^ d^−1^ ± standard deviation	Methodology	References
Civitavecchia, Italy	January, 2023	8–10	NCP	2.11 to 47.57; average 7.67 ± 5.92		*This study*
			GCP	3.52 to 79.29; average 12.79 ± 9.87		
			Rd	− 31.71 to − 1.41; average − 5.12 ± 3.95		
Magalluf Bay (Mallorca Island, Spain)	March 2001–October 2002	7	NCP	− 24.7 ± 7.0 to 88.8 ± 6.4	Dissolved oxigen concentration measurements	^49^
			GPP**	24.7 ± 5.3 to 173.3 ± 13.4		
Bay of Revellata (Corsica, France)	October, 2011	4	NCP	14.75 ± 5.36; 49.96 ± 11.13; 61.67 ± 24.46	Water samples spectrophotometry	^50^
Bay of Calvi (Corsica, France)	November to April, 1978 to 2014	10	NPP***	22.93 ± 1.72*****	Harvest method (Bay, 1984)	^51^
Alcanada (Mallorca Island, Spain)	Winter, 2012	4	NPP***	2.74	Dissolved oxygen concentration measurements	^52^
			GPP**	7.84		
			RR****	− 5.1		
Elba Island (Italy)	May, 2016	13	GPP**	151.4 ± 41.8 and 203.7 ± 50.6******	Eddy covariance instrument	^53^
			RR****	66.4 ± 18.3 and 91.8 ± 22.6******		
Bay of Revellata (Corsica, France)	November to December, 2006 to 2016	10	NCP	40 ± 15	Optodes measurements	^54^
Vroulia Bay (Lipsi Island, Greece)	Autumn, 2018 to Spring, 2019	7	GPP**	up to 60*******	PME miniDOT loggers	^55^
			NCP	up to 7*******		
			RR****	up to 50*******		

From a spatial point of view, the PP-SDM results showed very heterogeneous values between the depths of 8 m and 10 m, making it challenging to identify specific trends. The survey area is heavily anthropized, with multiple sources of disturbance to the marine environment (e.g., wastewater discharges, fishing, tourism, recreational boating, and sports activities), and their effect on the *P. oceanica* distribution and PP are difficult to assess considering present results. Anthropogenic activities affect the seagrass ecosystem distribution and functionality^56^ both locally with direct impacts [e.g.,^57^] and at broader spatial scales with indirect impacts [e.g.,^58^]. In our case study, to evaluate the influence of anthropogenic activities on *P. oceanica* distribution and PP in detail, further in situ data acquisition will be necessary, considering the current environmental conditions (e.g., seawater temperature and nutrient concentrations, light, and turbidity levels) and for longer periods of time.

In conclusion, this study focused on applying an integrated approach to map coastal benthic habitats linking USV surveys, ROV video acquisitions, and in situ* P. oceanica* ecosystem functioning measurements. This approach proved valuable in gathering detailed information about fragmented *P. oceanica* meadows in heavily anthropized coastal areas with multiple sources of disturbance.

The coastal area of northern Latium was thoroughly explored by unmanned mapping of bottom morphology, type, height, and coverage of *P. oceanica*. Data obtained through USV, coupled with functional measurements using benthic chambers, enabled the creation of a preliminary *P. oceanica* PP-SDM in the study area.

Future perspectives involve enhancing modelling data with a greater quantity of in situ observations to make them reliable for all seasons and the entire *P. oceanica* habitat. Further refinement of the proposed approach through the integrated application of multiple approaches will provide valuable information for a deeper understanding of coastal habitats. This information will be crucial for validating predictive numerical models focused on preserving ecosystem health, addressing restoration effort, and ensuring the sustainable use of marine resources.

## Methods

### Study area

**Figure 4:**
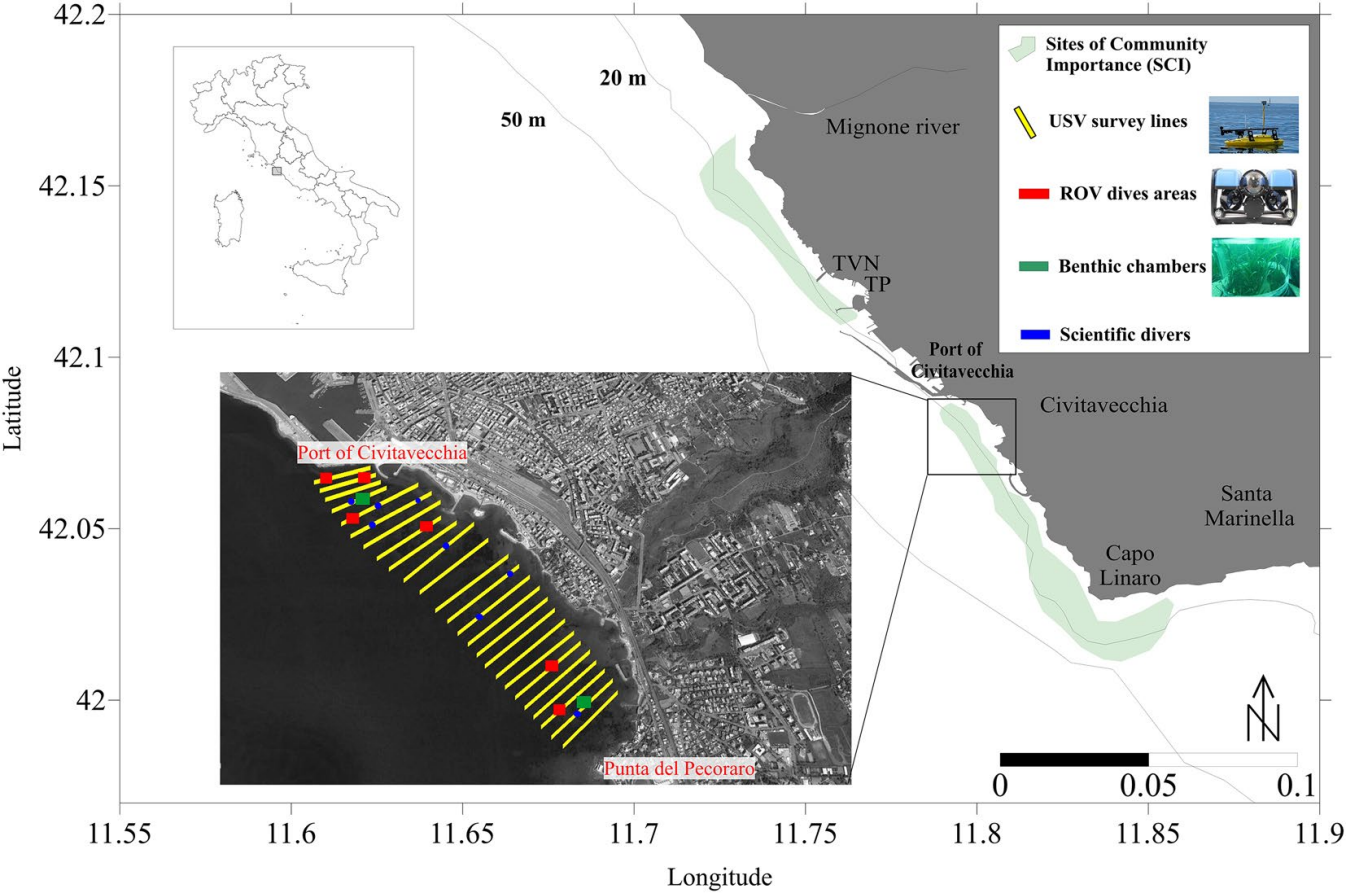
Study area (Northern Latium coast, Italy) and survey plan displayed in geographical coordinates. The survey plan was represented on a satellite image using the Google Earth Pro software (7.3 version, https://www.google.com/intl/it/earth/about/versions/).

The city of Civitavecchia is on the northern Latium coast (42°5.5454’N 11°47.7485’E; Northern Tyrrhenian Sea, Italy), about 70 km north of the city centre of Rome (Fig. 4). The coastal zone simultaneously hosts areas of high ecological value and important industrial realities. It benefits from the presence of two Sites of Community Importance (SCI; Habitat Directive 92/43/EEC; European Commission, 1992), characterized by *Posidonia oceanica* meadows and priority species such as *Corallium rubrum* and *Pinna nobilis*^3^, respectively located to the north and to the south of the Port structure.

The distribution of *P. oceanica* in this area appeared generally heterogeneous, with a strongly fragmented and discontinuous distribution pattern^59^.

The Port of Civitavecchia extends to the northwest of the city. It features regular cargo ferry piers, carrier ship docks ("Ro-Ro"), and cruise traffic making it a significant hub for maritime activities^60^.

Beyond the Port, the municipality of Civitavecchia hosts national strategic structures, including the gas-fueled combined-cycle power plant "Tirreno Power" (TP) and the coal-fired "ENEL Torrevaldaliga Nord" power plant (TVN)^61^, located at the northwest end of the Port.

### USV survey plan, data acquisition, validation, and analysis

Coastal benthic habitats mapping was carried out by analysing the data acquired along coast-to-offshore survey lines (Fig. 4). The data were collected south of the Port of Civitavecchia in a single day during the winter season (January 2023). A total of twenty five survey lines (average length of 430 m) were established from a depth of 1 m to 20 m, beginning approximately 60 m north of the Port and extending southward until about 150 m from Punta del Pecoraro. The survey lines bathymetric range has been selected considering existing *P. oceanica* distribution data^59^.

Surveys were carried out using a USV (EchoBoat, Seafloor Systems Inc., Shingle Springs, CA, USA) equipped with a single beam echosounder (MX Aquatic Habitat Echosounder, BioSonics Inc., Seattle, WA, USA). The USV was set in autonomous mode, navigating at a speed of 0.75 m/s, with a delay of 60 s between each survey line. The echosounder transducer (204.8 kHz of frequency, 9 degrees conical of beam angle) operated with a pulse length of 0.4 ms and 5 pings/s. This echosounder allows us to simultaneously acquire bottom depth, submerged aquatic vegetation height and coverage, and bottom type data^62–64^. BioSonics Visual Acquisition software was used for echosounder data acquisition, and BioSonics Visual Aquatic software and Surfer 9 (Golden Software Inc., USA) were employed for analysis, producing maps with information on bottom depth (m), bottom type, *P. oceanica* coverage (%), and height (m). The Kriging gridding method, incorporating all survey data, was applied.

To validate the echosounder data (bottom type and *P. oceanica* height), ground-truth observations were conducted using a ROV (Bluerobotics BlueROV2; Bluerobotics Inc., Torrance, CA, USA; a total of six dives) and by scientific divers (a total of eight dives) in the survey lines area (see Fig. 4). Each dive returned visual information of an area of approximately 100 m^2^. Scientific divers measured canopy height using a metric rod at each dive site (three replicates per site; blue dots, Fig. 4). ROV dives, totaling 12 ROV videos, were performed in designated areas (Fig. 4) with the ROV moving along linear routes at a slow speed (< 0.3 m/s) and constant height from the bottom (< 1 m). The ROV was equipped with an underwater acoustic tracking position system, depth and temperature sensors, and a compass.

To distinguish *P. oceanica* distribution from the rest of the submerged vegetation, a plant detection threshold of 0.2 m was applied during the echosounder data analysis^63^, based on the minimum height of *P. oceanica* leaves during the winter season as observed in ROV videos, images, and scientific divers' reports (see Supplementary Table 1). This was necessary due to the presence of algal winter coverage of similar height (e.g., genus *Asparagopsis*). Regarding the plant coverage, it is necessary to specify that this represents the number of pings above the plant detection threshold, considering a report interval of 10 pings.

Pearson’s correlation (r), linear regression, Root Mean Square Error (RMSE), and Cohen’s kappa coefficient were used to assess the reliability of *P. oceanica* height measurements (echosounder vs in situ observation). Cohen’s Kappa categories were described by authors in^36^. A significant level (p-value) of 0.05 was considered for each statistical test.

Where necessary, results were expressed using standard deviation as dispersion measure.

### *P. oceanica* ecosystem functionality assessment

Within the echosounder investigation area, measurements of *P. oceanica* ecosystem functioning were carried out using benthic chambers. Specifically, the chosen sites for placing the benthic chambers were near the breakwater of the Port of Civitavecchia at a depth of 8 m and close to Punta del Pecoraro at a depth of 10 m (green dots in Fig. 4).

The benthic chambers were customised for this research, consisting of transparent PVC cylinders (40 cm in diameter and 40 cm in height) with lids that can be easily positioned on submerged vegetation by scientific divers. At each site, the benthic chambers were placed haphazardly over *P. oceanica* and pushed into the sediment to a depth of 5 cm. A PVC skirt attached to the chamber’s base completely isolated it from the surrounding water. Each chamber was equipped with temperature, oxygen, and lux sensors (Atlas Scientific, www.atlas-scientific.com) to measure *P. oceanica* O_2_ fluxes. A magnetic pump facilitated water circulation within the chamber.

*Posidonia oceanica* meadows were incubated for measurements of Respiration (Rd) and Net Community production (NCP). Respiration was measured by covering the chambers with total black tissue, which was later removed to measure NCP. All incubations lasted for 90 min. To estimate gross community primary production (GCP), each Rd measurement was added to its corresponding NCP measurement (NCP +|Rd|= GCP)^65^. Each measure was expressed as millimoles of Oxygen per square meter per day (mmol O_2_ m^−2^ d^−1^) ± standard deviation.

At the end of in situ incubation, the density of *P. oceanica* and the length of each shoot within the chamber were measured (see Supplementary Table 2). The data acquired through the benthic chambers were analysed to identify the relationships existing between *P. oceanica* morphological and metabolic measured Rd, NCP, and GCP values by applying regression models. Finally, based on the best fit obtained, selected statistical models were applied to the echosounder *P. oceanica* height and density coverage dataset to retrieve a preliminary distribution model of the potential functionality of *P. oceanica* between the depths of 8 and 10 m in the studied area.

### Supplementary Information

Supplementary Table 1.

Supplementary Table 2.

## Data Availability

The bottom depth, plant height and coverage data are reported in Supplementary Table 1. The *P. oceanica* number of shoots and length are reported in Supplementary Table 2. Other information and additional data related to this work are available from the corresponding author (D.P.) on a reasonable request.
